# Supplementary material for: In Vivo Assay of Cortical Microcircuitry in Frontotemporal Dementia: A Platform for Experimental Medicine Studies
Source: Cereb Cortex. 2019 Jun 19;31(3):1837–47. doi: 10.1093/cercor/bhz024 (PMC7869085; doi:10.1093/cercor/bhz024)
Supplement: bhz024_SupplementaryMaterials [file bhz024_supplementarymaterials.docx]

Supplementary Table T1

| **Parameter [prior]** | | | **μ * exp(0)** | **σ** |
| --- | --- | --- | --- | --- |
| Extrinsic Connections [mV] | | | | |
| Forward | SP➞SS | | 200 | 1/16 |
|  | SP➞DP | | 100 | 1/16 |
| Backward | DP➞SP | | 200 | 1/16 |
|  | DP➞II | | 100 | 1/16 |
| Intrinsic Connections (Canonical Microcircuit) [mV] | | | | |
| Excitatory | | SP➞SS | 800 | 0 |
|  | | SS➞II | 800 | 0 |
|  | | DP➞II | 400 | 0 |
|  | | SS➞SP | 800 | 0 |
|  | | SP➞II | 800 | 0 |
|  | | SP➞DP | 800 | 0 |
| Inhibitory [ & gain, G] | | SS➞SS [G] | 800 | 0 |
|  | | II➞SS | 800 | 0 |
|  | | II➞II [G] | 800 | 0 |
|  | | SP➞SP [G] | 800 | 1/8* |
|  | | II➞DP | 400 | 0 |
|  | | DP➞DP [G] | 200 | 0 |
|  | | II➞SP | 800 | 0 |
| Time Constants [ms]  $K$ | | SS | 2 | 1/8 |
|  |  | SP | 2 | 1/8 |
|  |  | II | 10 | 1/8 |
|  |  | DP | 20 | 1/8 |
| Delays [ms] | | Extrinsic | 16 | 1/64 |
|  |  | Intrinsic | 1 | 1/64 |
| Spatial [observer] model | | | | |
| Contributing States | | SS SP II DP | 20% 80% 0% 20% | 1/8 1/8 0 1/8 |
| Electrode Gain | | All sensors | 64 | 1 |

Supp Material:

**Population specific coupling:**

Stellate cells:

$$S_{e}=S_{SP}$$

$$H=G_{SS\to SS}S_{SS}-G_{II\to SS}S_{II}-G_{SP\to SS}S_{SP}$$

$$U=1\left( A1only \right)$$

Superficial layer pyramidal cells:

$$S_{e}=S_{DP}$$

$$H=G_{SS\to SP}S_{SS}-G_{SP\to SP}S_{SP}-G_{II\to SP}S_{II}$$

Inhibitory interneurons:

$$S_{e}=S_{DP}$$

$$H=G_{SS\to II}S_{SS}+G_{DP\to II}S_{DP}-G_{II\to II}S_{II}+G_{SP-II}S_{SP}$$

Deep layer pyramidal cells:

$$S_{e}=S_{SP}$$

$$H=G_{DP\to DP}S_{DP}-G_{II\to DP}S_{II}+G_{SP-DP}S_{SP}$$
